# Implementation of Patient-Centered Education for Chronic-Disease Management in Uganda: An Effectiveness Study

**DOI:** 10.1371/journal.pone.0166411

**Published:** 2016-11-16

**Authors:** Trishul Siddharthan, Tracy Rabin, Maureen E. Canavan, Faith Nassali, Phillip Kirchhoff, Robert Kalyesubula, Steven Coca, Asghar Rastegar, Felix Knauf

**Affiliations:** 1 Division of Pulmonary and Critical Care, Johns Hopkins University, Johns Hopkins School of Medicine, Baltimore, Maryland, United States of America; 2 Department of Medicine, Yale School of Medicine, Yale University, New Haven, Connecticut, United States of America; 3 Global Health Leadership Institute, Yale University, New Haven, Connecticut, United States of America; 4 College of Health Sciences, Makerere University, Kampala, Uganda; 5 Department of Surgery, University Hospital Basel, Basel, Switzerland; 6 Department of Nephrology, Mt. Sinai Hospital, Mount Sinai School of Medicine, New York, New York, United States of America; 7 Department of Nephrology, Friedrich-Alexander-Universität Erlangen-Nürnberg, Erlangen, Germany; Universite de Bretagne Occidentale, FRANCE

## Abstract

**Background:**

The majority of non-communicable disease related deaths occur in low- and middle-income countries. Patient-centered care is an essential component of chronic disease management in high income settings.

**Objective:**

To examine feasibility of implementation of a validated patient-centered education tool among patients with heart failure in Uganda.

**Design:**

Mixed-methods, prospective cohort.

**Settings:**

A private and public cardiology clinic in Mulago National Referral and Teaching Hospital, Kampala, Uganda.

**Participants:**

Adults with a primary diagnosis of heart failure.

**Interventions:**

PocketDoktor Educational Booklets with patient-centered health education.

**Main Measures:**

The primary outcomes were the change in Patient Activation Measure (PAM-13), as well as the acceptability of the PocketDoktor intervention, and feasibility of implementing patient-centered education in outpatient clinical settings. Secondary outcomes included the change in satisfaction with overall clinical care and doctor-patient communication.

**Key Results:**

A total of 105 participants were enrolled at two different clinics: the Mulago Outpatient Department (public) and the Uganda Heart Institute (private). 93 participants completed follow up at 3 months and were included in analysis. The primary analysis showed improved patient activation measure scores regarding disease-specific knowledge, treatment options and prevention of exacerbations among both groups (mean change 0.94 [SD = 1.01], 1.02 [SD = 1.15], and 0.92 [SD = 0.89] among private paying patients and 1.98 [SD = 0.98], 1.93 [SD = 1.02], and 1.45 [SD = 1.02] among public paying patients, p<0.001 for all values) after exposure to the intervention; this effect was significantly larger among indigent patients. Participants reported that materials were easy to read, that they had improved knowledge of disease, and stated improved communication with physicians.

**Conclusions:**

Patient-centered medical education can improve confidence in self-management as well as satisfaction with doctor-patient communication and overall care in Uganda. Our results show that printed booklets are locally appropriate, highly acceptable and feasible to implement in an LMIC outpatient setting across socioeconomic groups.

## Introduction

Greater than 80% of non-communicable disease (NCD) related deaths occur in low- and middle-income countries (LMICs) [1]. The vast majority of these deaths are attributable to cardiovascular diseases, cancer, chronic respiratory conditions, and/or diabetes which are related to four largely modifiable risk factors–tobacco, unhealthy diet, physical inactivity and alcohol [1]. Beyond the human toll, the economic burden of NCDs is expected to be crippling for LMICs, potentially reversing all gains that have been achieved by poverty reduction efforts [2]. It is estimated that $47 trillion in global economic output will be lost due to NCDs by 2030 and, thus, that the cost of inaction would exceed that of proposed interventions for NCDs [2]. As a consequence, chronic disease management has been identified by the WHO as a key priority and “best buy” for combating the epidemiologic transition to NCDs in LMIC settings [1–3].

Patient-centered education is an essential component in chronic disease management [4]. Defined as a partnership between health care providers, patients, and families, patient-centered education provides patients with the information necessary to participate in medical decision-making. Medical care that is sensitive to patient dignity has been demonstrated to enhance information sharing between all members of a treatment team [4, 5]. Patient-centered education has been shown to a) foster communication [4], b) improve medication adherence [6, 7], c) decrease hospitalization [6], and d) affect positive changes in health habits for patients with chronic diseases in high-income settings [8, 9]. Among hospitalized patients, those who understand their discharge instructions—including how to take their medications—are 30% less likely to be readmitted [6]. Numerous studies have documented inadequate patient-centered care in high-income settings [4, 5, 9, 10]. Patient discontent has been demonstrated even when doctors considered communication adequate [10]. Poor communication between patients and providers has been associated with adverse health outcomes [11]. Although a number of single-center studies have shown low levels of patient satisfaction with care and communication in LMICs, as well as the potential for improved health outcomes, limited data exists regarding patient-centered communication in these settings [7, 12–14].

Public health interventions aimed at combating NCDs will require novel approaches as well as implementation of strategies which have been previously validated in high-income settings [15]. We describe the implementation of a previously validated patient-centered health tool among an outpatient population with primary diagnosis of heart failure in Uganda. Uganda is representative of many LMIC, as it is undergoing a transition in the relative burden of communicable to non-communicable diseases [16]. We examined patient-centered care among those with heart failure, as it is a disease process requiring complex health management with longitudinal follow up and has been previously studied to assess the role of doctor-patient communication in health outcomes [17, 18]. We assessed efficacy of this intervention in public and private clinical environments as well as adaptability, acceptability and feasibility of implementing patient-centered care in an LMIC setting.

## Materials and Methods

### Study Design, Setting and Participants

We conducted a mixed-methods, feasibility-implementation study to assess whether a patient-centered instrument, *PocketDoktor*, could be incorporated into patient counseling during outpatient visits for those with an established diagnosis of heart failure. We selected established patients, as these individuals had already undergone the necessary diagnostic testing and should have had some discussion of their diagnosis with a physician prior to enrollment in the study. The study location was the outpatient department of the Mulago National Referral and Teaching Hospital in Kampala, Uganda, which has over 400,000 outpatient visits annually amongst the specialty clinics [12]. To assess the role of socioeconomic factors in patient-centered care and satisfaction among patients with heart failure, we conducted sampling within an indigent patient population at the Mulago Outpatient Department (MOPD) Heart Failure Clinic, as well as among the outpatient clinic at the Uganda Heart Institute (UHI), a private-pay institution at the same physical location. While services are free at MOPD, UHI requires a consultation fee of 20,000 USh ($6), as well as fees for additional diagnostic testing and medications. This study was approved by the Yale University Institutional Review Board, Mulago Hospital Institutional Review Board and the Uganda National Council for Science and Technology. Written consent was obtained in English or Luganda for each participant and consent forms were approved by IRBs.

### PocketDoktor

*PocketDoktor* is a patient-centered education tool that facilitates provider and patient communication by describing diseases in short, illustrated booklets with accompanying text in layperson prose and associated pictograms. The booklets describe disease processes, diagnosis, treatment and long-term management. *PocketDoktor* contains interactive features that are designed to prompt patients to ask questions at each point of discussion, facilitating conversation about disease. To date the booklets have been evaluated for acceptability and satisfaction among European patients, showing high levels of satisfaction and an increase in self-reported disease-specific knowledge [19].

We integrated *PocketDoktor* booklets and health education within existing outpatient clinic visits at MOPD and UHI. The intervention consisted of a 20–30 minute appointment with a study health educator who went through the *PocketDoktor* booklets after the physicians discussed disease management with the patients. The health educator was trained in disease process, diagnosis and management of heart failure. The patient-centered education occurred in the same clinical space to facilitate discussion between patients, caregivers and providers. For those patients who were illiterate, the health educator utilized included pictograms to convey key concepts and engaged family members/caregivers in the discussion. Patients were given booklets to take home and encouraged to make note of questions for their next appointment.

### Formative Phase

A formative phase involving semi-structured interviews was conducted prior to initiation of the study with local providers and patients from the outpatient ward of Mulago Hospital. *PocketDoktor* booklets were translated from English to Luganda by certified translators. We partnered with three local physicians to rephrase complex medical terminology and redesign pictograms. Semi-structured interviews consisting of a convenience sample of 10 patients (equal distribution of males and females) and associated caretakers was conducted to assess adaptation, content, length and format. Comments were coded and content was updated accordingly. PocketDoktor booklets were back-translated for accuracy.

### Efficacy Trial

The efficacy trial consisted of a prospective-cohort study conducted between November 2014 and April 2015. Clinic physicians at UHI and MOPD were informed of the study on days of recruitment and were asked to refer all patients with a known diagnosis of heart failure. Inclusion criteria for the study included a primary diagnosis of heart failure (confirmed by echocardiography), plans for future follow up visits, and age greater than eighteen years. We defined heart failure as the presence of symptoms and signs of disease with objective evidence of cardiac dysfunction by echocardiography using the European Society of Cardiology Guidelines [20]. Exclusion criteria included lack of fluency in Luganda or English. Demographic information including gender, residence, occupation, income, level of education, literacy, marital status and self-reported health status was collected from those who consented.

### Outcome Measures

Quantitative questionnaires concerning patient activation and satisfaction as well as qualitative interviews were applied before and after intervention. The primary outcome of the feasibility study was change in level of patient activation after administration of *PocketDoktor* intervention. The Patient-Activation Measures (PAM-13) is a 13-item questionnaire designed to elicit responses from a person about his or her attitudes toward confidence, knowledge and ability to self-managing health and has been utilized to assess patient-centered care among those with chronic diseases [21]. The scale is based on the Guttman technique with items ordered according to level of difficulty and has been validated in numerous settings to assess doctor-patient communication and health outcomes [21–23]. We assessed patient activation in three domains (knowledge of condition, medical treatment options available, and ways to prevent further problems with health condition). Secondary outcomes included satisfaction with materials as well as satisfaction with doctor-patient communication. A patient satisfaction questionnaire, which was validated by Nabbuye-Skeandi et al. in the same hospital setting, was adapted for this study [12, 24]. The responses were recorded on a five-point Likert-type scale of response options, ranging from strongly disagree to strongly agree to elicit endorsement of a particular statement. In addition, we collected data concerning satisfaction with *PocketDoktor* materials, patient-physician communication and overall satisfaction with care. Semi-structured interviews were conducted examining the patient’s understanding of heart failure (including signs and symptoms, diagnosis and management), patient-doctor communication and overall satisfaction. Patients were followed up at their next appointment (3 months later, on average) and repeat questionnaires were administered and qualitative interviews were conducted.

### Data Analysis

Mean scores were calculated as a composite measure of satisfaction using each of the items in the general satisfaction indicator by clinic type with lower scores indicating greater satisfaction. Also, mean scores for the three dimensions of patient activation were calculated for each subject. In descriptive analysis frequencies and proportions were obtained for categorical variables, while means and standard deviations (SDs) were calculated for continuous variables. All associations were considered statistically significance at *P*-values of ≤0.05. Data were entered into Epidata software and analyzed using the STATA version 12.0.

Qualitative data was analyzed using grounded theory with a constant comparative approach [25]. Two separate investigators analyzed data (TS, FN), independently labelling concepts and generate codes based on the concepts until theoretical saturation was reached and no new codes were bring introduced in the interviews. Coded lines were then compared and applied based on group consensus among the investigators. This paper describes the results of the formative phase as well as the quantitative portion of the study; we anticipate disseminating the qualitative outcomes in a separate manuscript.

## Results

105 participants were referred to the health educator by the attending physician and all consented to participate in the study. 93 completed baseline and follow up surveys. Of those who were lost to follow up, 1 participant died and 11 did not follow up for the duration of recruitment. Participants with heart failure seen in the clinic were predominantly female (65% [n = 61]). 48% (n = 45) of those who were included in analysis were seen in the private clinic (UHI) while the rest were seen in the general public outpatient clinic (MOPD) (Table 1). Lower levels of formal employment were observed in the public clinics compared to private clinics. Participants in the public clinics stated lower levels of income compared to those seen in private clinics. In addition, those followed in the public clinics reported lower levels of education and literacy compared to those in the private setting. There were no significant differences in self-reported health status between public and private clinics, with the majority in both settings reporting poor or fair health status.

**Table 1:** Distribution of sociodemographic variable by clinic status (N = 93).

Variable	Private N = 49 (%)	Public N = 44 n (%)	p-value
**Gender**			0.388
Male	19 (38.8)	13 (29.6)	
Female	30 (61.2)	31 (70.4)	
**Occupation**			0.292
Employed	12 (24.5)	6 (13.6)	
Unemployed	25 (51.0)	29 (65.9)	
Self-employed	12 (24.5)	9 (20.5)	
**Average Monthly Income**			0.001
150,000USH	34 (69.4)	40 (90.9)	
150,000–500,000 USH	6 (12.2)	3 (6.8)	
500,000-1million USH	6 (12.2)	1 (2.3)	
1million-2million USH	1 (2.0)	0 (0.0)	
>2 million USH	2 (4.1)	0 (0.0)	
**Level of Education**			0.072
No study	7 (14.3)	10 (22.7)	
Primary	12 (24.5)	17 (38.6)	
Secondary	16 (32.7)	13 (29.6)	
University	14 (28.6)	4 (9.1)	
**Level of Reading/Writing**			<0.001
Illiterate	1 (2.0)	12 (27.3)	
Some reading/writing	24 (49.0)	23 (52.3)	
Fully literate	24 (50.0)	9 (20.5)	
**Marital Status**			0.196
Married	34 (69.4)	22 (50.0)	
Divorced	4 (8.2)	2 (4.6)	
Widowed	5 (10.2)	10 (22.7)	
Single	4 (8.2)	8 (18.2)	
Separated	2 (4.1)	2 (4.6)	
**Self-Reported health status**			0.203
Poor	17 (34.7)	19 (43.2)	
Fair	25 (51.0)	24 (54.6)	
Good	6 (12.2)	1 (2.3)	
Excellent	1 (2.0)	0 (0.0)	

N and column percent reported. P-values are from χ^2^ tests. Percentages may not sum to 100 due to rounding

Few participants stated strong baseline levels of satisfaction with doctor-patient communication and overall care in both public and private clinical settings at baseline, (2.3% [n = 1] and 28.6% [n = 14] respectively). Levels of satisfaction with doctor-patient communication and overall care were lower among those followed in public clinics compared to private settings (6.8% [3] vs 24.5% [12], p = 0.005 (Table 2).

**Table 2:** Distribution of baseline satisfaction items by clinic status (N = 93).

	Private N = 49	Public N = 44	p-value
**Satisfaction with patient education in Mulago**			<0.001
Extremely satisfied	14 (28.6)	1 (2.3)	
Moderately satisfied	32 (65.3)	27 (61.4)	
Neither satisfied or dissatisfied	0 (0.0)	2 (4.6)	
Moderately dissatisfied	3 (6.1)	12 (27.3)	
Extremely dissatisfied	0 (0.0)	2 (4.6)	
**I am satisfied that my doctor told me about my condition, the treatment options and how I can stay healthy.**			0.005
Strongly agree	12 (24.5)	3 (6.8)	
Agree	31 (63.3)	26 (59.1)	
Neither agree/disagree	1 (2.0)	0 (0.0)	
Disagree	5 (10.2)	15 (34.1)	
Strongly disagree	NA	NA	

### Patient Activation Measure Outcomes

At baseline participants in both private and public outpatient clinics reported low levels of patient activation in regards to their condition, treatment options, and ways to prevent exacerbation, though those seen in public outpatient settings reported lower levels compared to those in the private settings (Table 3). At follow up, participants in both clinic settings reported improvement in activation measure for each respective category (80% stated improvement in disease knowledge, 80% stated improved knowledge of treatment options, and 83% stated improved knowledge of ways to present worsening of condition, p<0.001). Greater improvement in activation measures were noticed among participants followed in public clinics versus private ones with 70.5% and 75.5% stating, respectively, that they strongly agreed with the statements, “I understand the nature and causes of my health condition.” (Tables 4 & 5).

**Table 3:** Mean baseline PAM 13 measures by clinic type (N = 93).

	Private Mean (SD)	Public Mean (SD)	p-value
I understand the nature and causes of my health condition	3.82 (0.93)	2.72 (0.95)	<0.001
I know the different medical treatment options available for my health condition	3.82 (1.03)	2.80 (0.95)	<0.001
I know how to prevent further problems with my health condition	4.02 (0.80)	3.32 (0.98)	0.003

p-values are from t-tests

**Table 4:** Change in PAM 13 measures from baseline to follow-up, stratified by clinic type.

	Private N = 49		Public N = 44	
	Change Mean (SD)	p-value	Change Mean (SD)	p-value
I understand the nature and causes of my health condition	0.94 (1.01)	<0.001	1.98 (0.98)	<0.001
I know the different medical treatment options available for my health condition	1.02 (1.15)	<0.001	1.93 (1.02)	<0.001
I know how to prevent further problems with my health condition	0.92 (0.89)	<0.001	1.45 (1.02)	<0.001

p-values are from χ^2^ test. PAM 13 scale (1–5)

**Table 5:** Baseline and follow-up proportion of respondents that strongly agree with PAM 13 measures by clinic type (N = 93).

Baseline	Private n (%)	Public n (%)	p-value
I understand the nature and causes of my health condition	9 (18.4%)	0 (0.0%)	0.003
I know the different medical treatment options available for my health condition	11 (22.5%)	0 (0.0%)	<0.001
I know how to prevent further problems with my health condition	11 (22.5%)	1 (2.3%)	0.004
**Follow-up**			
I understand the nature and causes of my health condition	37 (75.5%)	31 (70.5%)	0.583
I know the different medical treatment options available for my health condition	41 (83.7%)	32 (72.7%)	0.200
I know how to prevent further problems with my health condition	46 (93.9%)	34 (77.3%)	0.022

p-values are from χ^2^ test. PAM 13 scale (1–5)

### Appropriateness, Acceptability and Feasibility

*PocketDoktor* booklets were found to be locally appropriate and accepted through satisfaction surveys (Table 6). All participants expressed being satisfied to some extent with the education materials. They also felt that the writing and information had moderate or extreme clarity with the materials being at least moderately appealing and looking at least moderately professional. All respondents also reported being between moderately and extremely likely to recommend the contents of the educational materials. The intervention was feasible to implement in this setting with minimal burden to providers stated, although for the purposes of this investigation health education was provided by a study health educator. Participants reported that they were talked with about their disease and that pictures were used as part of the explanation. They noted that this information generated questions and they would talk to friends and family about the materials.

**Table 6:** Distribution of satisfaction with educational materials at follow-up by clinic type (N = 93).

	Private N = 49	Public N = 44	p-value
	n (%)	n (%)	
**Satisfaction with education materials**			0.085
Extremely satisfied	43 (87.8)	32 (72.7)	
Moderately satisfied	6 (12.2)	11 (25.0)	
Slightly satisfied	NA	1 (2.3)	
Neither satisfied or dissatisfied	NA	NA	
Slightly dissatisfied	NA	NA	
Moderately dissatisfied	NA	NA	
Extremely dissatisfied	NA	NA	
**Clarity of writing and information on the educational materials**			0.704
Extremely clear	46 (93.9)	40 (90.9)	
Moderately clear	3 (6.1)	4 (9.1)	
Slightly clear	NA	NA	
Not at all clear	NA	NA	
**Visual appeal of educational materials**			0.222
Extremely appealing	48 (98.0)	40 (90.9)	
Very appealing	1 (2.0)	3 (6.8)	
Moderately appealing	NA	1 (2.3)	
Slightly appealing	NA	NA	
Not at all appealing	NA	NA	
**Professionalism of look and feel of the educational materials**			0.058
Extremely professional	44 (89.8)	32 (72.7)	
Very professional	5 (10.2)	11 (25.0)	
Moderately professional	NA	1 (2.3)	
Slightly professional	NA	NA	
Not at all professional	NA	NA	
**Likelihood of recommending the content of the educational materials to others**			0.363
Extremely likely	46 (93.9)	38 (86.4)	
Very likely	3 (6.1)	5 (11.4)	
Moderately likely	NA	1 (2.3)	
Slightly likely	NA	NA	
Not at all likely	NA	NA	

p-values are from Fishers exact test

## Discussion

In this mixed-methods study we examined the effect of patient-centered health education on health activation measures among those with heart failure in outpatient settings in Uganda and assessed the implementation of the *PocketDoktor* booklets with regards to appropriateness, acceptability and feasibility of intervention. Furthermore, we described concrete differences between public and private clinical settings, adding to the understanding of patient preferences between different payer groups. While a number of studies have examined the effects of patient-centered care in high-income settings, this is one of the first examining patient-centered care in a low-income setting and informs strategies for chronic disease management in other LMICs.

Similar to prior studies, we found low levels of baseline satisfaction with care and patient-physician communication among patients in our setting [12]. An understanding of patient confidence in self-management is a critical step in assessing the role of a patient-centered intervention in health outcomes. While no authors have examined patient-activation in LMIC settings, one study found significant improvements in self-reported medication adherence and blood pressure among a private paying population of Nigerian patients with a similar intervention, consistent with our findings [14]. Our study differed from previous ones by specifically examining the efficacy of our intervention in relation to patient-centered outcomes, as well as feasibility of implementing the intervention among mixed-payer populations. While a significant portion of the health-seeking population in LMIC settings is indigent, the private health care market is substantial, and understanding differences between groups will be critical for effective systemic strategies to manage NCDs [26].

Patient activation is a complex outcome including patient literacy, socioeconomic status and patient-doctor communication [27, 28]. Lower levels of patient activation were observed among participants seen in public clinics, consistent with the differences seen between public and private patients in the patient activation component factors. Intervention with *PocketDoktor* resulted in greater improvement in activation measures among this group, possibly as a result of the pictograms that allowed for use of the tool with patients that have limited literacy, though these results may also represent a ceiling effect, as private paying patients had higher baseline scores. Additionally the improvement in activation scores across domains may be a consequence of a social acceptability response bias which have been noted in previous studies in the region. The intervention was found to be locally appropriate and feasible. The average time of administration was 15 minutes by the end of the study and was well received by patients and providers alike. Of note, for the purposes of this study the intervention required the addition of a dedicated health educator. Further research will need to be conducted to assess the implementation of this model by existing members of the healthcare team (e.g. physicians, nurses, or community health workers). Given the relative low-cost of booklets, the intervention has the potential to be scaled with limited resource burden.

Limitations of this study included the study setting. Mulago National Referral and Teaching Hospital is a large tertiary hospital in the capital of Uganda. Although it is representative of other LMIC hospitals in regards to limited resources, inadequate ratio of physicians to patients, and long waits, the majority of patients with chronic disease typically receive care in rural health settings; 80% of Uganda is rural and patients typically receive care at local health centers or regional hospitals if at all [12, 29]. Additional investigations will need to examine the implementation of *PocketDoktor* at the regional and community levels. While this pilot aimed to assess efficacy and feasibility of implementation, larger trials assessing health outcomes could provide necessary data to assess cost-effectiveness of patient-centered care.

Given the rising burden of global NCD morbidity and mortality, LMIC health systems will need to address this epidemiologic transition from the angle of both chronic disease management and lifestyle-based shared risk factor reduction. Patient-centered approaches will be key components of any chronic-disease management strategy [3]. Interventions aimed at incorporating patient-centered NCD management within existing community-based health efforts, traditionally oriented towards infectious disease and malnutrition, may prove to be both cost-effective and transformative [30]. New technologies such as m-health (mobile phone-based health technology) may allow for implementation of patient-centered care directly in rural communities [31, 32]. As with any new health system intervention, the ideal program should be low-cost, require minimal input of new resources, culturally acceptable, and scalable.

## Conclusion

This feasibility-implementation study marks an initial step toward implementing patient-centered education to address chronic illness in Uganda. Although the numbers are small, this data also suggests that a patient-centered education strategy may even have a greater impact on those patients who are most socially or economically disadvantaged; these are the same individuals who, together with their families, are likely to bear the greatest burden of the looming NCD crisis.

## Supporting Information

S1 File PocketDoktor Booklet Luganda. (PDF)

S2 File Study Questionnaires. (DOC)

S3 File Baseline and Follow-up Data. (XLSX)
