# Supplementary material for: Implementation of Patient-Centered Education for Chronic-Disease Management in Uganda: An Effectiveness Study
Source: PLoS One. 2016 Nov 16;11(11):e0166411. doi: 10.1371/journal.pone.0166411 (PMC5112982; doi:10.1371/journal.pone.0166411)

Tegeera Omubiri Gwo

# Endwadde Y'Okunafuwa Omutima

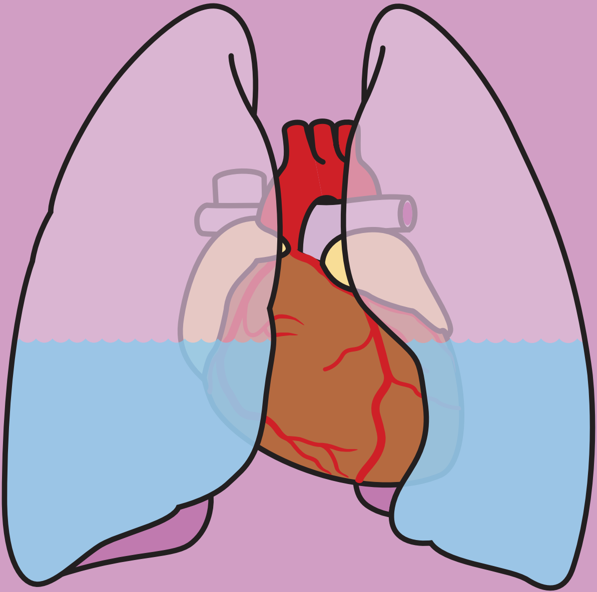

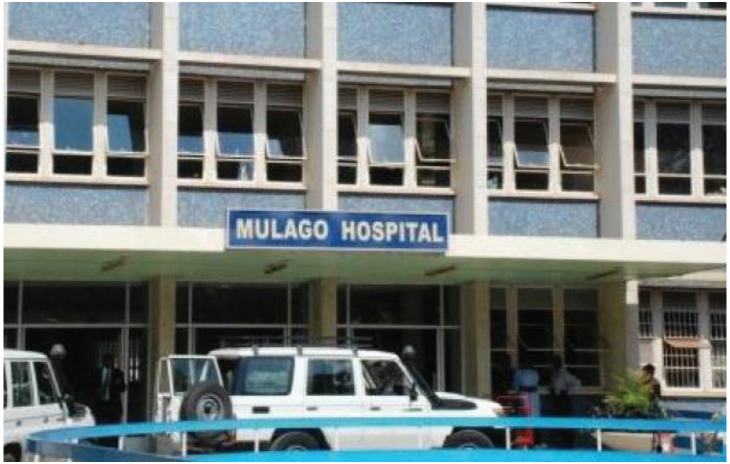


Ekitongole kye'ddagala

Mu ddwaaliro e'mulago

0414554008/1

(RASHOTS office)





## Abemikwano, abalwadde baffe, abe'famire za'balwadde, wamu n'abalabiririra abalwadde,

Akatabo kano kakoleddwa abasawo okuva mu ttendekero e makerere ne ddwaliiro lya mulago nga bali wamu na basawo okuva mu ttendekero lya yale mu amerika, nga tusuubira akatabo kano kajja kukuyamba okutegeera obulungi ekikulwaza nengeri gyoyinza okubeerawo n'obulwadde.

Oweereddwa akatabo kano lwakuba olina obulwadde bw'omutima omunafu. Akatabo kano kajja kusobozesa okutegeera obulungi biki ebibaawo ssinga omutima gunafuwa, engeri y'okwejjanjabisa n'engeri gy'oyinza okwekuuma nga oli mulamu wamu n'okwetangira embeera okusajjuka.

Teweerabira kubuuza bibuuzo...

# Omutima Gusangibwa Wa ?

Omutima gusangibwamu mu kifuba era osobola okuguwulira ku luuyi lwo olw'akkono olwe'kifuba kyo. Omulimu gwagwo omukulu kutambuza musaayi mu mubiri, ekisobozesa ebitundu by'omubiri ebirala okusigala nga biramu.

Kyamugaso nnyo okukuuma omutima nga mulamu kisobozeseze ebitundu byo'mubiri ebirala okusigala nga biramu,



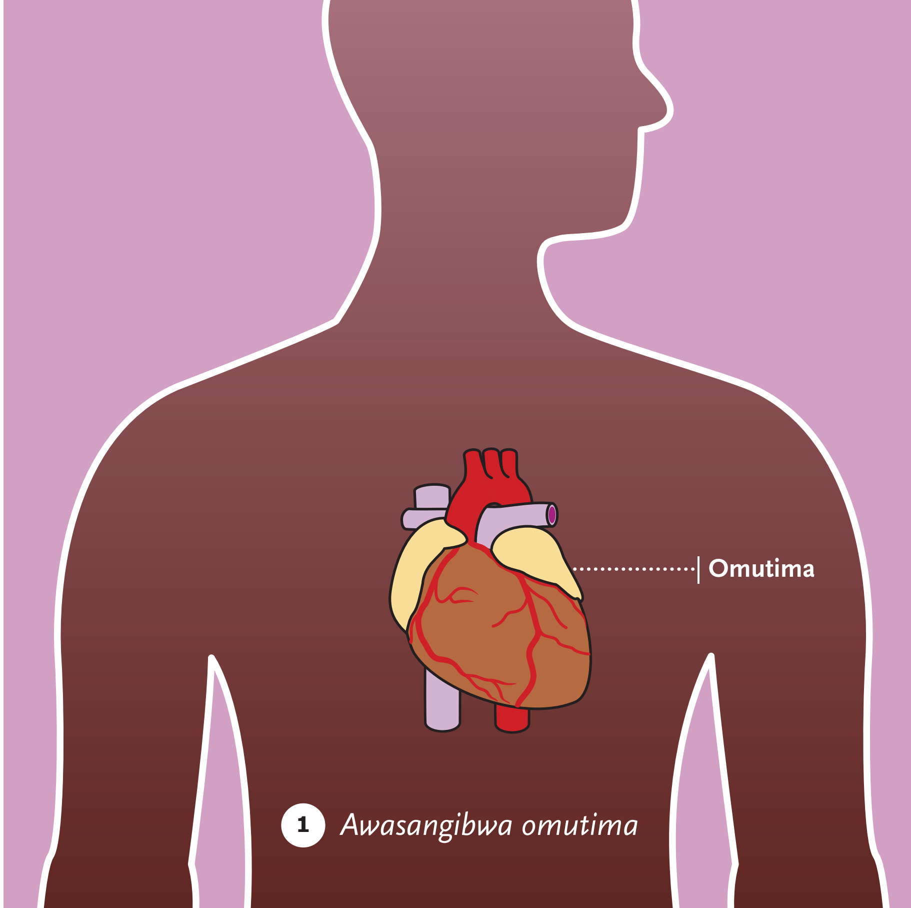

# Omutima Gukola Ki ?

Omutima gulina enjuyi bbiri era ga gupika omusaayi okuva mu mawuggwe nga gulimu empewo yo'bulamu wamu ne'biriisa negutwalibwa mu bitundu by'omubiri ebirala. Ate obujji obuggala enjuyi ezo bufuga entambula yomusaayi mu mutima.

- Oluuyi olwa ddyo lutwala omusaayi okuva mu mubiri negutwalibwa mu mawuggwe.
- Oluuyi olwa kkono lutwala omusaayyi okuva mu mawuggwe nga gulimu empewo y'obulamu negutwalibwa mu bitundu ebirala nebisobola okufuna empewo y'obulamu.

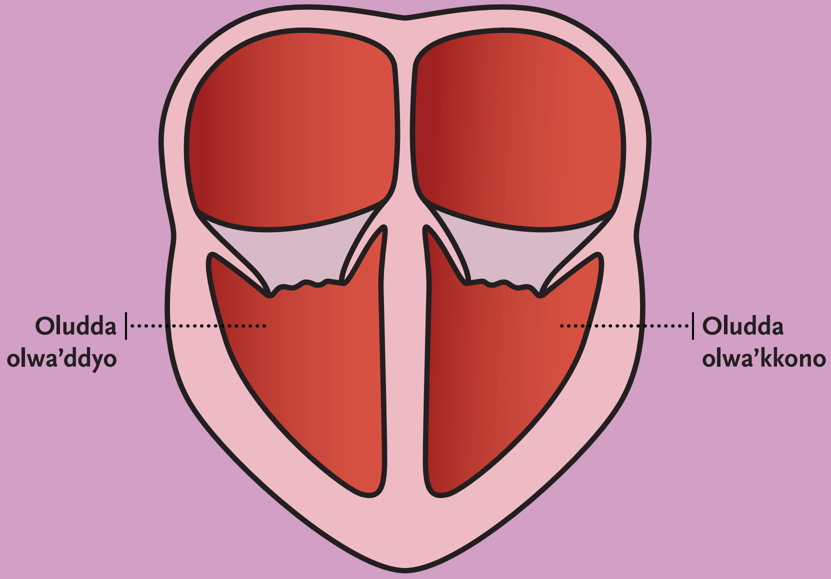


2 *Enkola y'omutima*

# Omutima Okunafuwa Kyeki ?

Omutima okunafuwa kitegeeza omutima guba tegukola bulungi, era guba tegusobola kupika musaayi mu bwangu obweetaagisa.

Ssinga ekizibu kibeera ku luuyi olw'akkono, amazzi gajjuza amawuggwe.

ISsinga ekizibu kibeera ku luuyi olwa ddyo, amazzi gajjuza ebitundu byo'mubiri ebirala.

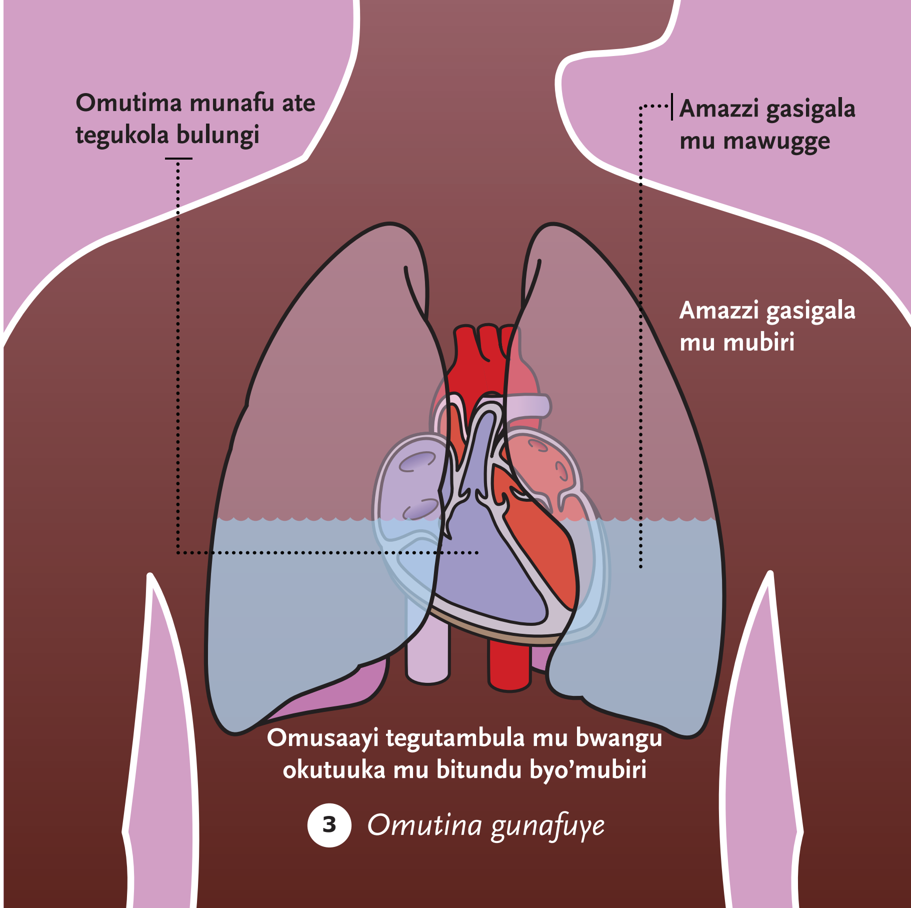


3 *Omutina gunafuye*

## Kiki Ekireetera Omutima Okunafuwa ?

Ensonga emu ekunafuya omutima eva ku kuzibikira emisuwa egiriikiriza omutima, kireetera omutima okunafuwa nekuugejja.

Obulwadde bwa puleesa buleetera omutima okukola ennyo ekitali kya bulijjo, kino nakyo kinafuya omutima.

Mu balwadde abamu obujji bwo mutima buyinza okugaana okwegaala oba okweguula.

Ebiseera ebimu obuwuka bwa virus nabwo bunafuya omutima, omwenge omungi, obunene,n'okunywa segereeti.

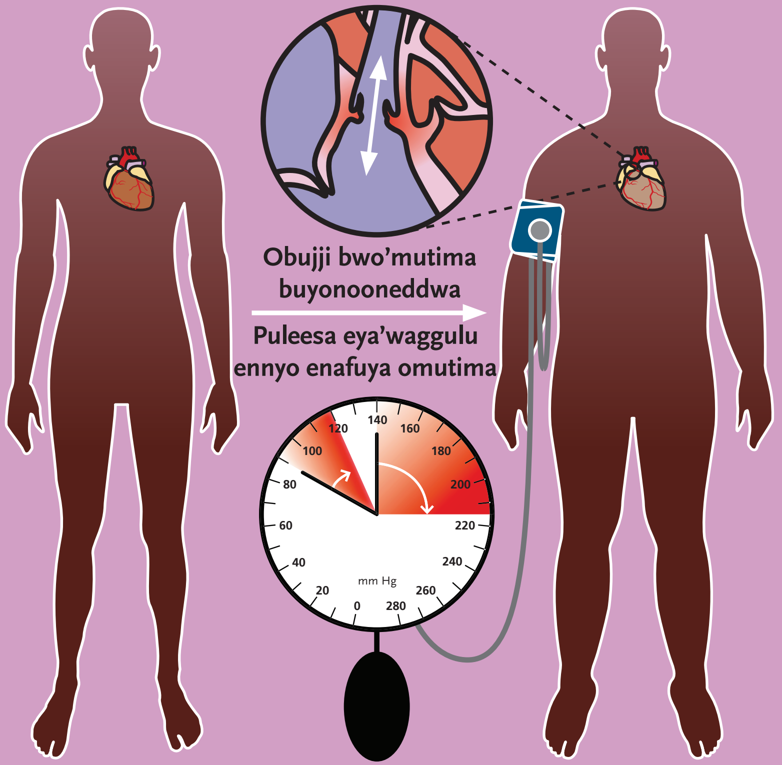


4 *Ebireetera omutima okunafuwa*

## Bubonero Ki Kwolabira Nti Omutima Gunafuye ?

Okunafuwa omutima kireeta obubonero bungi,naye abalwadde abasinga obungi bafuna obuzibu mu kussa. Obuzibu mu kussa bubaawo ssinga amazzi gagenda mu mawuggwe nga mangi. Era nga ebiseera ebisinga omuntu ayinza n'okukolola.

Omutima okunafuwa kiyinza okuleeta kantolooze ssinga omusaayi guba mutono ogutuuka ku bwongo.

Amazzi bwegagenda mu bitundu by'omubiri ebiralaa, oyinza okuzimba ebigere n'obukongovvule. Kiyinza n'okuleeta amazzi mu lubuto. Oyina okufuuyisa emirundi mingi ekiro osobole okwejjako amazzi amangi mu mubiri.

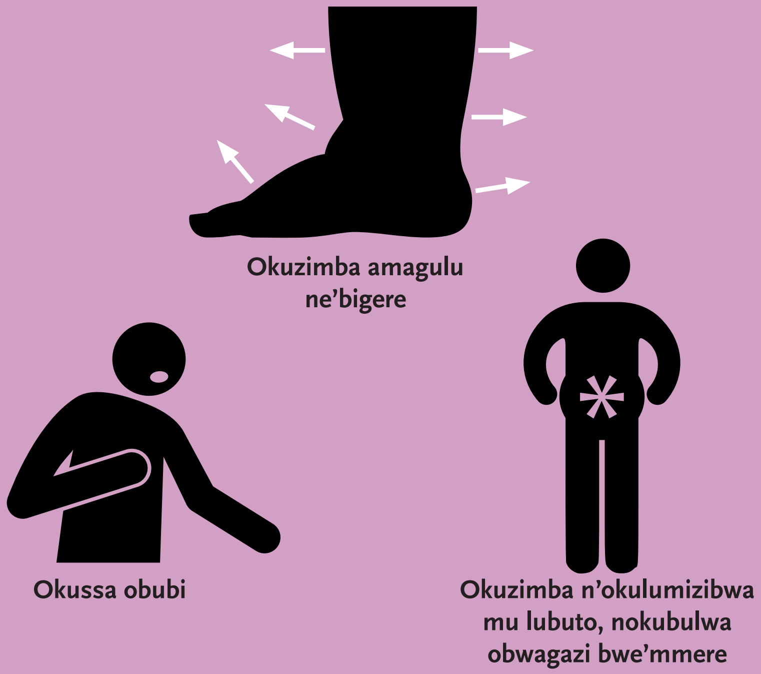


5 *Obubonero bw'omutima ogunafuye*

# Omutima Okunafuwa Kuzuulibwa Kutya ?

Okuzuula obulwadde buno kwesigamizibwa nnyo ku biki by'ogambye omusawo, obubonero n'okukeberebwa no'musawo.

Mu ngeri yemu, ekifananyi kyekifuba kiraga amazzi amangi agali mu mawuggwe.

Ekyuma ekiyitibwa sikaani kisobola okulaga ebinywa by'omutima wamu n'enkola yagwo.



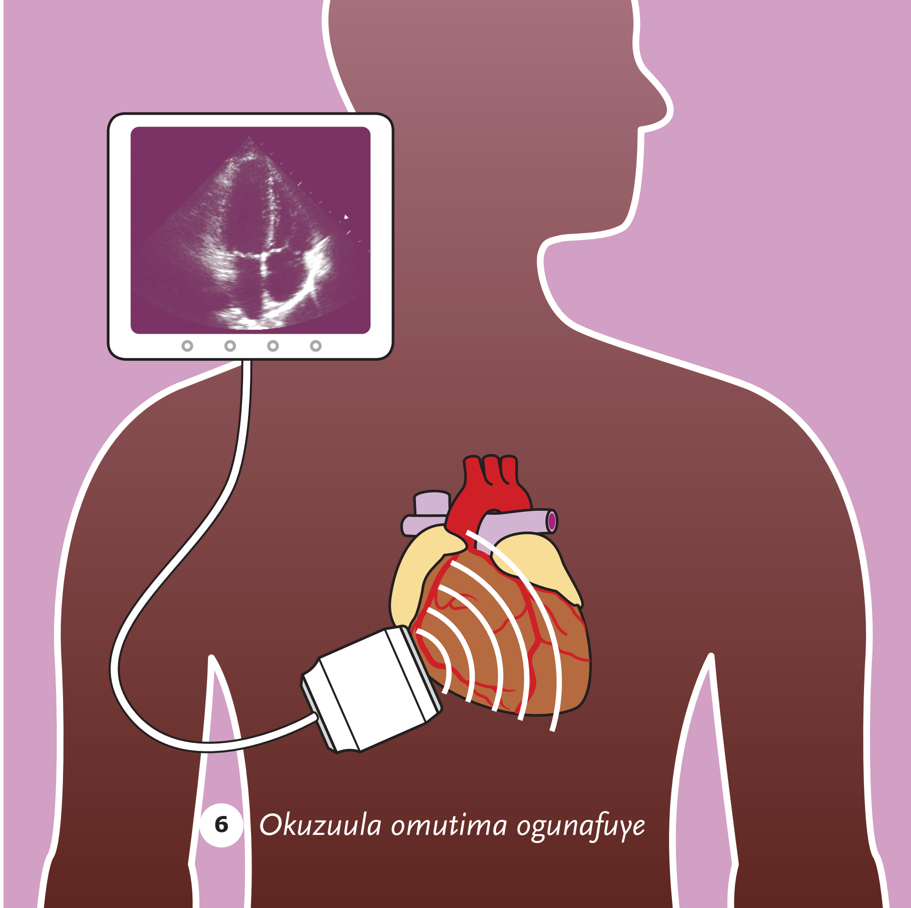

6 *Okuzuula omutima ogunafuye*

# Okunafuwa Omutima Ku Jjanjabibwa Kutya ?

Amadagala agakozesebwa okujjanjaba okunafuwa omutima mulimu gano wa mmanga.

- Amadagala agaddiriza emisuwa ne gassa puleesa wansi, mu ngeri eyo negakendeeza omulimu gw'omutima.
- Amakerenda agongera obwagazi bw'okufuuyiisa gayamba ensigo okufulumya amazzi n'omunnyo oguyitiridde mu mubiri.
- Amakerenda gakendeeza puleesa n'entunnunsi.

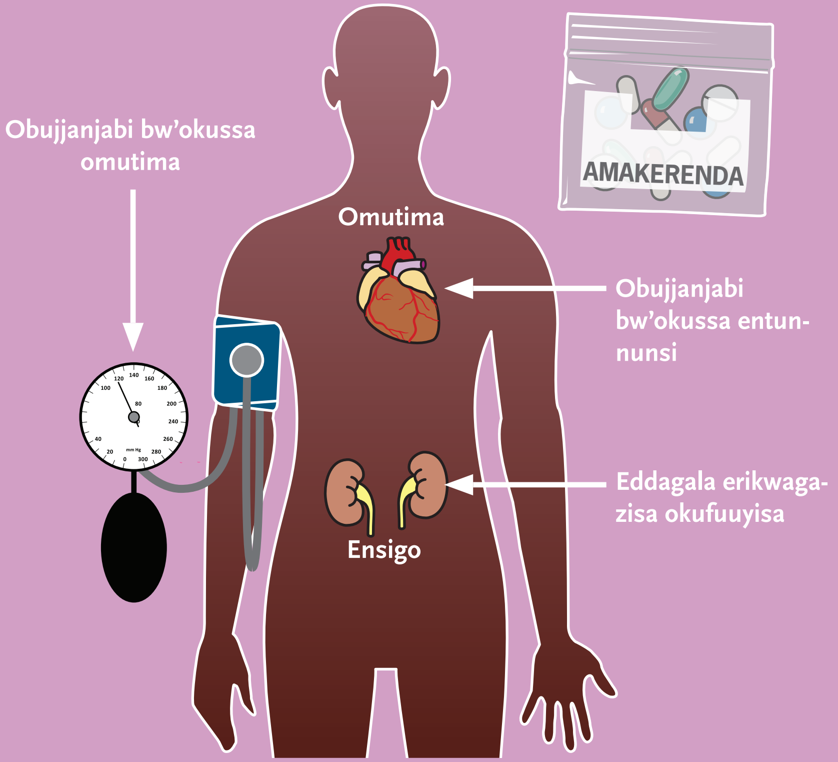


7 *Okujjanjaba omutima omunafu*

# Nkyuusa ntya endya n'e nnywa yange ?

Endya ennungi erimu omunnyo mutono n'amasavu matono. Weewale emmere empakire n'okwongera omunnyo omungi mu mmere gy'ofumba

Kubaganya ebirowoozo n'omusawo wo ku bungi bw'amazzi goyina okunywa, okunywa ennyo kiyinza okwongera okusajjula embeera era oyinza okulagibwa okunywa obutasukka liita emu ne kindu bulu lunaku.

Lekaayo omwenge kubanga gusajjula obunafu bwomutima.



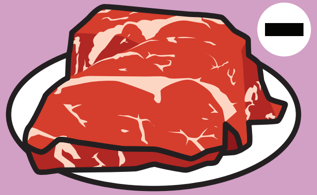

Wewale emmere eyamasavu

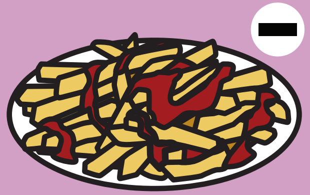

Tolya mmere nnyinyike mu butto

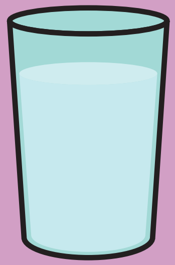

Kendeeza ebyokunywa

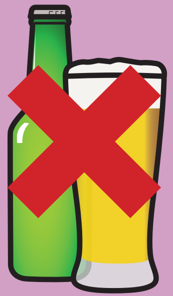

Tonywamwenge

# Tuunuulira nnyo obuzito wamu no'bunero

Bwoba olina minzaani, osobola okwepima buli kumakya, oba ssi ekyo, genda ku kalwaliro akakuli okumpi bakupime.

Tukubye akabox ku ddyo,wandiikamu obuzitto bwo buli lunaaku. Tegeeza omusawo wo ssinga weyongerako obuzito bwa kiro emu mu kiro, oba wakati wa kiro 2 ne 3 mu sabiiti ennamba

Wandiika wansi obubonero bwolina, buno bwayogeddwaako dda wa ggulu mu katundu akaatuumiddwa obubonero bw'o mutima ogunafuye.



| Emisana | Obubonero | Obuzito |
|---|---|---|
| 1 | | |
| 2 | | |
| 3 | | |
| 4 | | |
| 5 | | |
| 6 | | |
| 7 | | |
| 8 | | |
| 9 | | |
| 10 | | |
| 11 | | |
| 12 | | |
| 13 | | |
| 14 | | |
| 15 | | |
| 16 | | |
| 17 | | |
| 18 | | |
| 19 | | |
| 20 | | |
| 21 | | |
| 22 | | |
| 23 | | |
| 24 | | |
| 25 | | |
| 26 | | |
| 27 | | |
| 28 | | |
| 29 | | |
| 30 | | |
| 31 | | |

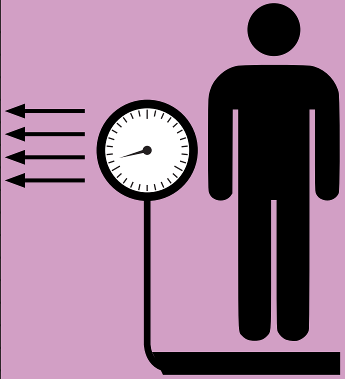

**Okweyongera obuzito obussuka kiro 1 buli lunaku, 2-3 kiro buli wiiki**

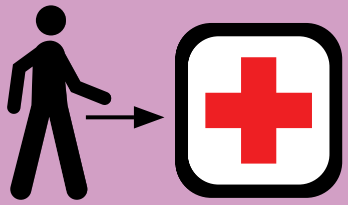

**9** *Weetegereze obuzito nobubonero bwo*

# Nyinza ntya okweyamba no bulwadde buno ?

Okunafuwa omutima buzibu obwalukonvuba. Newankubadde busobola okujjanjabirwa, naye tebugenda. Bulijjo mira eddagala lyo ate ogoberere namagezi omusawo gaakuwa.

Bulijjo kebera puleesa yo wamu n'obuzito bwoba nga olina ebikozesebwa, era tereka bulungi ebivuddemu.

Bwoba ofuweeta ssegereeti, muviireko ddala.

Okukola dduyiro kijja kuyamba omutima gwo nga mulamu. Naye nga tonaba kutandika dduyiro, sooka weebuuzeku musawo wo.

Okwebaka obulungi nakyo kyamugaso ssinga oba nga oli waakubeera mulamu.



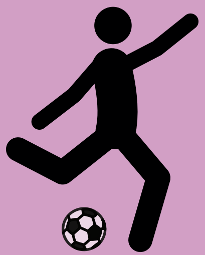
Totuula wamu

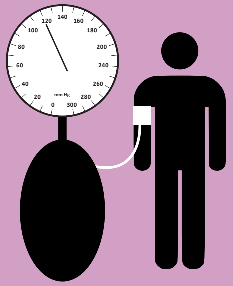
Fuga puleesa yo

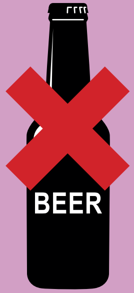

VVa ku bbiya

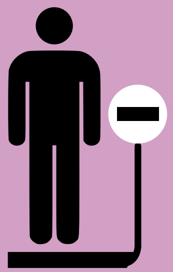
Fuga obuzito

**10** *Labirira omutima gwo*

# Ddi lwennina okunoonya obujjanjabi?

- Komawo mu ddwaaliiro ssinga ofuna bino wammanga.
- Okulumizibwa mu kifuba okwamanyi oba puleesa eri waggulu.
- Okussa obubi oba okuwunga.

Laba omusawo ssinga olaba nga embeera yo ebijja. Oyinza okufuna obubonero buno wammanga:

- Okweyongera okussa obubi n'okukolola.
- Okuzimba ebigere okuyitiridde, amagulu, olubuto, n'emikono.
- Okweyongera mu buzito(okussuka mu kiro emu olunaku oba 2-3 kiros mu wiiki emu)
- Omutima okuba nga gukubira kumu oba nga enkuba yagwo tegukwatagana

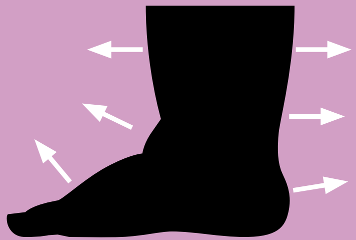

Okuzimba ebigere na magulu

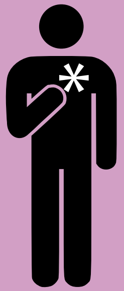

Okulumizibwa mu kifuba

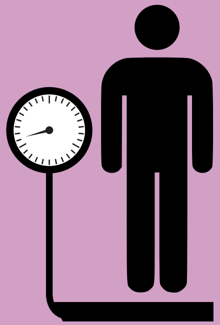

Okweyongera ennyo obuzito

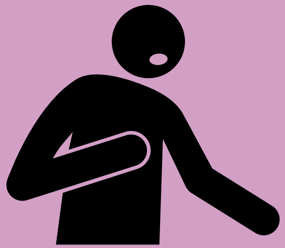

Okussa obubi

**11** *Obubonero obulabula*

# Ebibuuzo byo musawo wo:





POCKETDOKTOR

Okumanya ebisingawo n'okumanya kubikwata ku butabo bunowww.
pocketdoktor.com
or email to post@pocketdoktor.com

Akatabo kano tekakuwa Magezi ga kwejjanjaba. Ebiri mu katabo kano omuli ebifananyi wamu n'obubaka, Byakuyamba kutambuza bubaka. Era obubaka buno tebugendereddwa kubukozesa mu kifo kyo'musawo. Okwetuumira obulwadde, oba okwejjanjaba. Bulijjo funa amagezi okuva ew'omusawo wok u buli kibuuzo. Kyoyinza okubeera nakyo. Tosambajjanga amagezi agakuweereddwa omusawo wok u lwebintu by'somye. Mu katabo kano. Ssinga okizuula nga olina ekizibu ekyetaagisa obujjanjabi obwamangu, bulijjo tuukirira. Omusawo wo mu bunnambiro. Okwesigulira ku biri mu katabo kano, nogaana amageziagakuweereddwa abasawo. Okikola kululwo. Akatabo kano tekajja kuyimirirawo kunnyonyola butuufu, buwanvu,obwesigwa, oba obujja bwe biri mu. Katabo kano. Tewajja kubawo ngeri yonna aktabo kano lwekajja kunenyezebwa mu mateeka ku kukosebwa kwonna. Okunaaleetebwa oluvannyuma lwokukozesa akatabo kano.

1. Edition 2013
©2013 by Pocketdoktor GmbH
Authors: Felix Knauf, M.D.; Philipp Kirchhoff, M.D; Robert Kalyesubula, MBCh B; Steve Coca, M.D.; Tracy Rabin, M.D.; Irene Nakalema, MBCh B; Trishul Siddharthan, M.D. .
Design: Patrick Lane, Helmut Kraus (www.helm69.com)
Printed in USA

Tewali katundu kali mu katabo kano kakkirizibwa kukyusibwa, oba okwozebwamu mu ngeri yonna nga tofunye lukusa kuva mu bawandiisi.

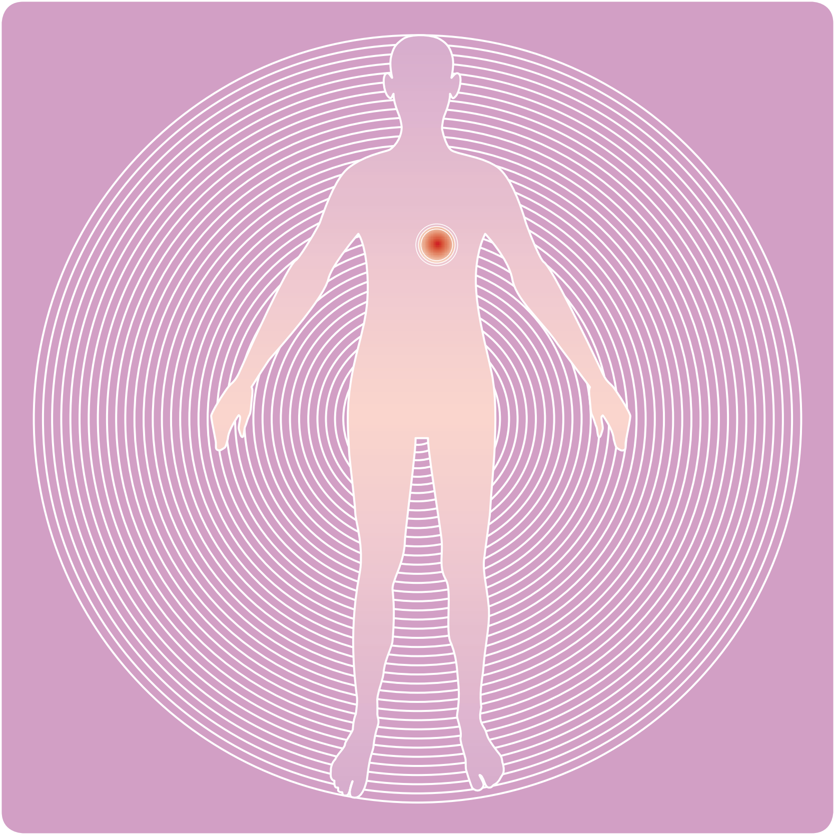

Supplement: S1 File — (PDF) [file pone.0166411.s001.pdf]
