# Supplementary material for: Implementation of Patient-Centered Education for Chronic-Disease Management in Uganda: An Effectiveness Study
Source: PLoS One. 2016 Nov 16;11(11):e0166411. doi: 10.1371/journal.pone.0166411 (PMC5112982; doi:10.1371/journal.pone.0166411)
Supplement: S2 File — (DOC) [file pone.0166411.s002.doc]

**Language_________________________**

**ACCEPTABILITY AND IMPLEMENTATION OF FACILITATED PHYSICIAN-PATIENT EDUCATION VIA POCKET DOKTOR AMONG PATIENTS WITH HEART FAILURE AT MULAGO HOSPITAL**

**RECRUITMENT INFORMATION**

**STUDY NUMBER:**

Demographic Data

Gender Male Female

Age

Home Address Kampala Out of Kampala

Occupation Employed Unemployed Employed

Average Income/Month <150,000USH 150-500,000USH 500,000-1M 1M-2M USH 2M

Level of Education No Study 1 Primary 2 Secondary 3 University

Level of Reading/Writing Illiterate Some Reading/Writing Fully Literate

Religion Catholic Protestant Islam Other

Tribe

Marital Status Married Divorced Widowed Single Separated

Self-reported overall health status Poor Fair Good Excellent

Date of recruitment

Heart Failure Background

Have you been educated on your condition and medications for your heart failure?

Yes No

Have you been educated about your heart failure?

Yes No

Do you have an ECHO?

Yes No

If yes, what is the EF?

How many times have you been in the hospital in the last 1 month or the past 1 year?

**Satisfaction Questions**

1. Overall, how satisfied with patient education in Mulago?

|  Extremely satisfied 1   Moderately satisfied 2   Neither satisfied nor dissatisfied 3    Moderately dissatisfied 4   Extremely dissatisfied 5 |
| --- |

**2. I am satisfied that my doctor told me about my condition, the treatment options and how I can stay healthy.**

Strongly Agree 1

Agree 2

Neither Agree/Disagree 3

Disagree 4

Strongly Disagree 5

**3. I am satisfied that I understand the nature and causes of my health condition.**

Strongly Agree 1

Agree 2

Neither Agree/Disagree 3

Disagree 4

Strongly Disagree 5

**4. I am satisfied that I know the different medical treatment options available for my health condition.**

Strongly Agree 1

Agree 2

Neither Agree/Disagree 3

Disagree 4

Strongly Disagree 5

**5. I am satisfied that I know how to prevent further problems with my health condition.**

Strongly Agree 1

Agree 2

Neither Agree/Disagree 3

Disagree 4

Strongly Disagree 5

|  |
| --- |

**PAM-13**

1) I understand the nature and causes of my health condition.

Strongly Agree Agree Neutral Disagree Strongly Disagree

2) I know the different medical treatment options available for my health condition.

Strongly Agree Agree Neutral Disagree Strongly Disagree

3) I know how to prevent further problems with my health condition.

Strongly Agree Agree Neutral Disagree Strongly Disagree

**Qualitative Questions**

How do you feel when you have problems with your condition?

**Patient Knowledge**

1. Please describe what your doctor has told you is wrong with your body.
2. What other information would you like to know about your condition?
3. Please describe the treatment options for your health condition.
4. Please describe ways you can prevent further problems with your health condition.

**Outcomes Measures**

1. How many times did you have to go to the hospital in the past three months?
2. What medications are you currently on?

**Language_________________________ FOLLOW UP**

1. Overall, how satisfied with the educational materials?

|  Extremely satisfied 1   Moderately satisfied 2   Slightly satisfied 3   Neither satisfied nor dissatisfied 4   Slightly dissatisfied 5   Moderately dissatisfied 6   Extremely dissatisfied 7 |
| --- |
|  |

2. How clear is the writing and information on the educational materials?

| **** Extremely clear 1   Moderately clear 2   Slightly clear 3   Not at all clear 4 |
| --- |
| **3. How visually appealing are the educational materials?**  **** Extremely appealing 1  Very appealing 2   Moderately appealing 3   Slightly appealing 4   Not at all appealing 5 |

4. How professional is the look and feel of the educational materials?

| Extremely professional 1   Very professional 2  Moderately professional 3  Slightly professional 4   Not at all professional 5 |
| --- |

5. How likely are you to recommend the content of the educational materials to others?

|  Extremely likely 1   Very likely 2   Moderately likely 3  Slightly likely 4  Not at all likely 5   1. Did someone sit down with you and talk about your disease?   □ Yes □ No □ Not sure   1. Did someone use any pictures to help you explain your disease?   □ Yes □ No □ Not sure   1. Did these pictures and explanations help you to ask questions you may have?   □ Yes □ No □ Not sure   1. Did reading the materials stimulate you to ask any questions of your doctor?   □ Yes □ No □ Not sure   1. Would you talk about the materials with your family or friends?   □ Yes □ No □ Not sure PAM-13 |
| --- |

1) I understand the nature and causes of my health condition.

Strongly Agree Agree Neutral Disagree Strongly Disagree

2) I know the different medical treatment options available for my health condition.

Strongly Agree Agree Neutral Disagree Strongly Disagree

3) I know how to prevent further problems with my health condition.

Strongly Agree Agree Neutral Disagree Strongly Disagree

**Qualitative Questions**

Patient Satisfaction

- 1. How do you feel about your communication with your doctor?
  2. How has the educational material improved communication with you and your doctor?

Patient Knowledge

1. Please describe your medical conditions.
2. What are some ways doctors treat your condition
3. Please describe some ways you can prevent further problems with your health condition.

Outcomes Measures

1. How many times did you have to go to the hospital in the past three months?
2. What medications are you currently on?
3. How many times have you had symptoms from your disease?
   1. What were those symptoms?
